# Health Promoting Bioactive Properties of Novel Hairless Canary Seed Flour after In Vitro Gastrointestinal Digestion

**DOI:** 10.3390/foods9070932

**Published:** 2020-07-14

**Authors:** Emily Mason, Lamia L’Hocine, Allaoua Achouri, Mélanie Pitre, Salwa Karboune

**Affiliations:** 1 Saint-Hyacinthe Research and Development Centre, Agriculture and Agri-Food Canada, 3600 Casavant Boulevard West, Saint-Hyacinthe, QC J2S 8E3, Canada; emily.mason2@mail.mcgill.ca (E.M.); allaoua.achouri@canada.ca (A.A.); melanie.pitre@canada.ca (M.P.); 2 Department of Food Science and Agricultural Chemistry, Macdonald Campus, McGill University 21, 111 Lakeshore, Ste-Anne-de-Bellevue, QC H9X 3V9, Canada; salwa.karboune@mcgill.ca

**Keywords:** canary seed, digestate, bioactive peptide, antihypertensive, antidiabetic, antioxidant

## Abstract

The bioactive properties and health-promoting effects of two novel yellow (C09052, C05041) and two brown (Calvi, Bastia) hairless canary seed (*Phalaris canariensis* L.) cultivars were investigated in comparison to two common cereal grains (wheat and oat). The cereal flours were digested using the standardized INFOGEST in vitro human gastrointestinal digestion model. The three-kilo dalton molecular weight cutoff (3 kDa MWCO) permeate of the generated digestates was assessed in vitro for their antioxidant, chelating, antihypertensive and antidiabetic activities. The results showed no significant differences in studied bioactivities between yellow and brown canary seed cultivars, except for antioxidant activity by the DPPH and chelating Fe2+ assays, where brown cultivars had higher activities. Canary seeds had superior or equivalent antioxidant activity than those from oat and wheat. The anti-hypertensive activity (Angiotensin-converting enzyme (ACE) inhibition) in yellow canary seed cultivars was significantly higher than that of oat and wheat, particularly for C09052 and Calvi varieties. Peptides exhibiting the highest antihypertensive activity from the permeate of the C09052 canary seed variety were further fractionated and identified by mass spectrometry. Forty-six peptides were identified belonging to 18 proteins from the Pooideae subfamily. Fourteen of the parent proteins were homologous to barley proteins. Peptides were analyzed in silico to determine potential bioactivity based on their amino acid composition. All 46 peptides had potential anti-hypertensive and anti-diabetic activities and 20 had potential antioxidant activity, thereby validating the in vitro assay data. Canary seed peptides also exhibited potential antiamnestic, antithrombotic, immunostimulating, opioid and neuro-activity, demonstrating important potential for health promoting effects, particularly against cardiovascular disease.

## 1. Introduction

Hairless (glabrous) canary seed (*Phalaris canariensis* L.) is a novel true cereal grain which belongs to the family Poaceae, along with other prevalent cereal grains, such as wheat, oat, barley and rye [1]. Canary seed is produced primarily in Canada, which is the world’s largest producer (60% of world production) and first exporter of canary seed with over 75% market share [2,3]. The hairy seeds could only be used as birdseed since they were lined with inedible hair-like silica fibers that are deemed hazardous to human health as they were found to be causing lung damage and even esophageal cancer [4]. The Crop Development Center at the University of Saskatchewan in Canada developed a new ‘hairless’ or ‘glabrous’ canary seed from the hairy variety which is safe for human consumption and utilization by the food industry as a new cereal grain canary seed [5]. Hairless brown and yellow canary seed have received in 2016 novel food approval from Health Canada [1], as well as GRAS (generally recognized as safe) status from the U.S. Food and Drug Administration [6]. Due to its high protein content (19–21%) [7], this new edible cereal grain is emerging as an alternative source of plant proteins. The overall quality of a protein depends not only on its digestibility and amino acid bioavailability, but also the health promoting bioactive properties it demonstrates once digested [8,9]. It was reported that the biologic activities of cereals have mostly been attributed to the presence of glucans and polyphenols [10]. However, proteins from cereal (wheat, barley, oat) and pseudo-cereals (buckwheat, amaranth) are also known for producing bioactive peptides with health promoting effects when digested [9]. They have demonstrated a variety of bioactivities, including antioxidant, antihypertensive, antibacterial, antithrombic, anticancer, antidiabetic, immunomodulating and opioid activity, which depends on the source protein, amino acid composition and sequence [11,12]. Finding new sources of good quality plant proteins, other than the traditional sources, remains essential to meet the global growing demands for protein [8,9]. In terms of digestibility, several studies indicate that hairless canary seeds make an excellent addition or supplement to conventional animal feed, as it promotes growth, but also enhances protein digestibility [13,14,15,16]. For human digestibility of canary seed proteins, no in vivo study has been reported in the literature. Abdel-Aal et al. [17] used a multienzyme approach with trypsin, chymotrypsin and peptidase and established an in vitro protein digestibility of 84% in hairy canary seeds.

Some regions of the world—including Mexico—have utilized hairy canary seed as a traditional folk medicine to help combat chronic societal diseases including cardiovascular disease and diabetes [18]. Until recently, no scientific evidence proved the effect of these claims, but studies have since been conducted on hairy canary seed (*Phalaris canariensis* L.) proteins demonstrating antioxidant [19], antidiabetic [18] and antihypertensive activity [18,19] due mainly to the presence of bioactive peptides. These studies were conducted on hairy varieties of canary seeds, and although the nutritional value is comparable to the hairless varieties, the bioactivity of the newly approved hairless Canadian seeds remains unknown. Therefore, in a first step of this study the potential health promoting effects associated with the newly developed Canadian hairless yellow and brown canary seed cultivars were evaluated in comparison to commonly consumed cereal grains, through in vitro assessment of the antioxidant, chelating, antihypertensive and antidiabetic activities of the low molecular weight (<3 kDa) components of the gastrointestinal digestates of cereal flours. In a second step, the canary seed peptides that were responsible for the most promising antihypertensive bioactivity were further fractionated and identified by mass spectroscopy. Identified peptide sequences were also analyzed in silico to determine their potential bioactivities.

## 2. Materials and Methods

### 2.1. Materials

Four hairless dehulled canary seed (*Phalaris canariensis* L.) cultivars (two yellow cultivars C09052 and C05041 (now registered as cultivar Cibo), and two brown Calvi and Bastia cultivars) were used in this study. The canary seed cultivars were developed and produced by the Crop Development Center of the University of Saskatchewan (Saskatoon, SK, Canada). Oat (*Avena sativa*) cultivar (Turcotte) and one wheat (*Triticum estivum* L.) cultivar (snowbird) were purchased from Semican (Princeville, QC, Canada) and also used for comparison purpose in this study. All seeds were hand-cleaned to remove any broken seeds or foreign material.

Phosphate buffered saline (PBS), sodium bicarbonate (NaHCO3), monosodium phosphate (NaH2PO4), hydrochloric acid (HCl), sodium chloride (NaCl) and Tris-HCl were purchased from BioShop (Burlington, ON, Canada). 2,2′-Azobis(2-amidinopropane) dihydrochloride (AAPH), trolox, fluorescein, 2,2-diphenyl-1-picrylhydrazyl (DPPH), 2,2′-azino-bis(3-ethylbenzothiazoline-6-sulfonic acid) diammonium salt (ABTS), potassium persulfate (K_2_S_2_O_8_), ferrous chloride (FeCl_2_), ferrozine, borax (Na_2_B_4_O_7_·10H_2_O), angiotensin converting enzyme (ACE) from rabbit lung (A6778), N-Hippuryl–His–Leu hydrate (HHL) substrate (H1635), hippuric acid, trifluoroacetic acid (TFA), gly–pro p-nitroanilide hydrochloride (G0513), and mixed standards for the size-exclusion chromatography analysis (aprotinin., vitamin B12, Gly–Gly–Gly and carnosine) were all purchased form Sigma (St. Louis, MO, USA). Dimethyl sulfoxide (DMSO), ethanol and methanol were obtained from Fisher Scientific (Fair Lawn, NJ, USA). The dipeptidyl peptidase IV enzyme from porcine kidney (CD26, 317640) was purchased from Millipore Sigma (Burlington, MA, USA). Deionized water (Millipore) was used in all experiments.

### 2.2. Proximate Analysis of Cereal Flours

Canary seeds, oat and wheat flours were prepared by grinding frozen dehulled seeds into fine powders using an analytical mill (IKA A11, IKA, Staufen, Germany). The flours were stored at room temperature in the dark until used for analysis. Total nitrogen content of canary seed, oat and wheat flours were determined using a Vario MAX Cube (Elementar, Langenselbold, Germany), following the Dumas combustion method [20] with EDTA as a nitrogen standard. Crude protein content of cereal flours was calculated as total nitrogen multiplied by a conversion factor of 5.7 for canary seed and 6.25 for oat and wheat. Fat content was determined using a Soxtec apparatus (Foss Tecator Soxtec System HT-6, 1043 extraction unit, Brampton, OT, Canada) according to AACC method 30–25.01 [21]. Ash content was determined according to AACC official method 08–03.01 [22], and moisture was determined by drying 2 g sample in a Fisher Isotemp vacuum oven (Fisher Scientific, Montreal, Qc, Canada) for 5 h at 100 °C [23]. Total carbohydrate comprising starch and fiber content was estimated by percent difference. All determinations were done at least in triplicate and average values were computed.

### 2.3. Preparation of Cereal Flour Digestates

The flour digestates were prepared according to the standardized INFOGEST in vitro digestion method of Minekus et al. [24]. In the oral phase of digestion, 1 g of cereal flour (canary seed, oat and wheat) was incubated for 2 min at 37 °C, pH 7.0, with simulated salivary fluid (SSF) (1:1) containing α-amylase from porcine pancreas (75 U/mL of digestate). Then, the mixture was diluted (1:1, *v/v*) with simulated gastric fluid (SGF) containing pepsin from porcine gastric mucosa (2000 U/mL digest). The pH of the mixture was adjusted to pH 3.0 and then incubated for 2 h at 37 °C. Intestinal phase was carried out by diluting the mixture (1:1, *v/v*) with simulated intestinal fluid (SIF) containing pancreatin from porcine mucosa (100 U trypsin activity/mL digestate) and bile (10 mM). The pH of the mixture was adjusted to pH 7.0 and incubated for 2 h at 37 °C. The reaction was stopped by placing the solutions on ice and adding 1-mM AEBSF (protease inhibitor). The final digestates were centrifuged and the supernatants filtered through an Amicon 3-kDa molecular weight cutoff (MWCO) ultrafiltration unit (Millipore, Burlington, MA). Protein content in the 3K permeate was quantified by the Pierce BCA protein assay kit (Thermo Scientific, Waltham, MA, USA) using bovine serum albumin as a standard. Filtered samples were frozen at −80 °C until used for analysis.

### 2.4. Characterization of the Molecular Weight Distribution of the 3K Permeates of Cereal Flours Digestates

Size-exclusion chromatography (SEC-HPLC) was carried out according to the modified method of Achouri et al. [25]. The collected 3K permeates were further separated using an Enrich SEC-70 column (10 × 300 mm) (Bio-Rad Laboratories, Mississauga, ON, USA) connected to an Agilent-1200 Series HPLC system (Agilent Technologies Canada, Inc., Mississauga, ON, USA). Samples from the 3-K permeate (2.5 µL) were loaded on the column and eluted with 10-mM phosphate buffered saline, with 154-mM NaCl (pH 7.4) at a flow rate of 0.5 mL/min at 220 nm. Mixed standards comprising aprotinin (6.511 kDa), vitamin B12 (1.355 kDa), Gly–Gly–Gly (tripeptide, 189.17 Da) and carnosine (dipeptide, 226.23 Da) were mixed and used to estimate the molecular weight distribution.

### 2.5. Determination of Total Polyphenol Content (TPC) in the 3K Permeates of Cereal Flours Digestates

The total polyphenol content (TPC) in the dehulled flours and in 3K permeates of cereal flour digestates was determined using the Folin–Ciocâlteu reagent according to the modified method of Singleton and Rossi [26]. Ferulic acid, prepared in 70% ethanol containing 1% concentrated HCl, was used to generate the standard curve at concentrations between 0 to 500 mg/L. The TPC content in the samples was determined from the ferulic acid standard curve, and results were expressed as mg ferulic acid equivalents (FAE) per g sample for the undigested flours and/or per g of protein in the 3K permeates.

### 2.6. Determination of the Antioxidant and Chelation Activities

#### 2.6.1. Oxygen Radical Absorption Capacity (ORAC) Assay

The experimental work was carried out according to the method of Tomer et al. [27], with modification by Garrett, Murray, Robison and O’Neill [28]. AAPH (2,2′-azobis(2-amidino-propane) dihydrochloride; 79.65 mM) was prepared fresh in 75-mM phosphate buffer (pH 7.4). A fluorescein stock solution (1.2 µM) was prepared in advance with 10-mM PBS and stored at 4 °C, protected from light. The fluorescein working solution (0.96 nM) was prepared fresh from the fluorescein stock solution using 75-mM phosphate buffer (pH 7.4). Trolox, prepared in 75-mM phosphate buffer (pH 7.4) containing 5% DMSO, was used to generate the standard curve (6.25–100 µM). Twenty-five microliters of samples (digestates), standards, and blanks were loaded onto a black, clear bottomed 96-well microplate. A total of 150 µL of 0.96-nM fluorescein was added to each well and then incubated for 30 min at 37 °C. After the incubation period, 25 µL of 79.65-mM AAPH was injected into each well using an automatic injector and the fluorescence monitored using a Synergy HTX fluorescence reader (Bio-Tek, Winooski, VT) for 120 min at 37 °C with an excitation and emission wavelength of 485 nm and 520 nm, respectively (20 nm bandpass). The AUC (area under the curve) was calculated by the Bio-Tek software from the following equation:

(1) $$\mathrm{AUC}=\frac{R1}{R2}+\frac{R2}{R1}+\frac{R3}{R1}+\ldots \frac{Rn}{R1}$$

where *R*1 is the initial reading and *Rn* is the last reading taken. The Net AUC is calculated by subtracting the AUC of the blank from the AUC of the sample or standard:

Net AUC = AUC_sample/standard_ − AUC_blank_ (2)

The standard curve is obtained by plotting the Net AUC of the trolox dilution samples against their respective concentrations. The results are expressed as µmol of trolox equivalents (TE) per mg of protein in the 3K permeate.

#### 2.6.2. DPPH Free-Radical Scavenging Capacity Assay

The free radical scavenging activity was measured using the free radical DPPH (2,2-difenil-1-picrilhidrazil, Sigma Chemical Co, St. Louis, MO, USA) according to Orona-Tamayo et al. [29], with modification. Briefly, an aliquot of hydrolysate was diluted at different protein concentrations and 100 µL of diluted samples were added to a 96-well flat bottom microplate, followed by 100 µL of 0.05-mM DPPH in ethanol (stored at −20 °C). The plate was incubated for 30 min in the dark at room temperature, and the absorbance read at 517 nm using an Epoch microplate spectrophotometer (Bio-Tek, Winooski, VT, USA). The data were converted into a percentage of radical scavenging activity from the following equation:

DPPH inhibition (%) = 1 − (As/Ac) × 100%(3)

where As was the absorbance of the sample and Ac was the absorbance of ethanol without added inhibitor. The DPPH inhibition was plotted against its respective concentration to determine the IC_50_ value, the inhibitory concentration required to scavenge 50% of the DPPH radical, which was calculated by the Epoch software from a four-parameter logistic curve.

#### 2.6.3. ABTS Assay

The ABTS (2,2′-azino-bis(3-ethylbenzothiazoline-6-sulfonic acid) assay was carried out according to Re et al. [30], with modification by Chen et al. [31]. A 7-mM ABTS solution in water containing 2.45-mM potassium persulfate was prepared. The absorption of the ABTS+ working solution was adjusted to 0.700 ± 0.02 absorbance at 734 nm using 100% ethanol at room temperature. Fifty microliters of appropriately diluted samples were added to a 96-well flat bottom microplate, followed by 180 µL of ABTS+ working solution. After incubating the microplate for 6 min in the dark at room temperature, the absorbance was read at 734 nm using an Epoch microplate spectrophotometer. The data were expressed as a percentage of radical scavenging activity from the following equation:

ABTS inhibition (%) = 1 − (As/Ac) × 100%(4)

where As is the absorbance of the sample, and Ac refers to the absorbance of 50% ethanol without added inhibitor. The ABTS inhibition was plotted against its respective concentration to determine the IC_50_ value; the inhibitory concentration required to scavenge 50% of the ABTS cation was calculated by the Epoch software from a four-parameter logistic curve.

#### 2.6.4. Iron Chelating Activity (Fe^2+^) Assay

The assay was carried out according to the modified method of Orona-Tamayo et al. [29]. Fifty microliters of appropriately diluted sample concentrations were added to a 96-well flat bottom microplate, followed by 25 µL of 0.25-mM FeCl_2_ and 25 µL of 0.625-mM ferrozine (both prepared in water). After incubating the microplate for 10 min in the dark at room temperature, the absorbance was read at 562 nm using an Epoch microplate spectrophotometer. The data were expressed as a percentage of chelating activity from the following equation:

Fe^2+^ inhibition (%) = 1 − (As/Ac) × 100% (5)

where As is the absorbance of the sample, and Ac refers to the absorbance of water without added inhibitor. The Fe^2+^ inhibition was plotted against its respective concentration to determine the IC_50_ value (the inhibitory concentration required to scavenge 50% of the ferrozine –Fe^2+^ complex), which was calculated by the Epoch software from a four-parameter logistic curve.

### 2.7. Determination of the Antihypertensive Activity by ACE Inhibition Assay

The ACE (angiotensin-converting enzyme) inhibition activities were determined using the modified method of Barbana and Boye [32], with modification by Rui et al. [33]. Ten microliters of ACE enzyme (8 mU) was mixed with 10 µL of appropriately diluted hydrolysate (0–8 mg/mL) prepared in 100 mM borax buffer (pH 8.3) containing 300-mM NaCl and incubated at 37 °C for 10 min. Then, 50 µL of 1-mM hippuryl–His–Leu (HHL) substrate was added, and the reaction mixture was incubated for 30 min at 37 °C. The concentration of ACE enzyme (8 mU) was determined by performing preliminary enzymatic assays using various concentrations of the ACE enzyme (1 to 10 mU). The reaction was stopped by adding 85 µL of 1-M HCl. Five microliters of the mixture was injected into a 4.60 × 250 mm Aqua C18 column (5-µm pore size 125 Å, Phenomenex, Torrance, CA, USA) and the elution of hippuric acid (HA) and the consumption of HHL substrate were monitored at 228 nm. Samples were eluted with 50% (*v/v*) methanol in water containing 0.1% TFA at a flow rate of 0.5 mL/min for 15 min. The ACE inhibitory activity (%) was calculated using the following equation:

ACE inhibition (%) = 1 − (PAs/PAc) × 100%(6)

where PAs was the peak area of the sample and PAc was the peak area of the enzyme (ACE) and substrate (HHL) alone without the presence of hydrolysate peptide (inhibitor). The ACE inhibition was then plotted against its respective concentration to determine the IC_50_ value, the inhibitory concentration required to scavenge 50% of the ACE enzyme. The IC_50_ value was calculated from the equation of a four-parameter logistic curve.

### 2.8. Determination of the Antidiabetic Activity by DPP-IV Inhibition Assay

The DPP-IV (dipeptidyl peptidase IV) inhibition assay was carried out according to Velarde-Salcedo et al. [34], with modifications by Nongonierma and FitzGerald [35]. Twenty-five microliters of appropriately diluted digestates (0–5 mg/mL), prepared in 0.1-M Tris-HCl (pH 8.0), were added to 25 µL of 1000-uM Gly–pro-p-nitroanilide in a 96-well flat bottom microplate. The mixtures were incubated for 10 min at 37 °C, then, 50 µL of 0.005 U/mL DPP-IV enzyme (prepared in 0.1-M Tris-HCl, pH 8.0, [2.5 mU/mL final]) was added to each well and the microplate incubated at 37 °C for 1 h. After the incubation period, the absorbance was read at 415 nm using an Epoch microplate spectrophotometer. The percentage of DPP-IV inhibition was estimated as follows:

DPP-IV inhibition (%) = 1 − (As/Ac) × 100%(7)

where As is the absorbance of the sample, and Ac is the absorbance of water, enzyme and substrate alone without added inhibitor. The DPP-IV inhibition was plotted against its respective concentration to determine the IC_50_ value, the inhibitory concentration required to scavenge 50% of the DPP-IV enzyme. The IC_50_ value was calculated by the Epoch software from a four-parameter logistic curve.

### 2.9. Peptide Identification by Mass Spectroscopy and In Silico Analysis of Their Potential Bioactivity

Based on the results from the study on the bioactivities assessment, the C09052 3K permeate was fractionated by SE-HPLC and the separated fractions were recovered, lyophilized and retested for their ACE inhibition activity. The fraction exhibiting the highest ACE inhibition activity was analyzed by LC-MS for peptide identification. The lyophilized sample was first solubilized in 5% acetonitrile and 0.2% formic acid and then loaded on a C18 precolumn (0.3 mm × 5 mm) followed by separation on a reversed-phase column (150 μm × 150 mm) with a gradient from 10% to 30% acetonitrile and 0.2% formic acid at 600-nL/min flow rate for 56 min, using an Ultimate 3000 HPLC system (Eksigent, Dublin, CA) connected to an Q-Exactive Plus mass spectrometer (Thermo Fisher Scientific, San Jose, CA, USA). Each full MS spectrum acquired at a resolution of 70,000 was followed by 12 tandem-MS (MS-MS) spectra on the most abundant multiply charged precursor ions. Tandem-MS experiments were performed using collision-induced dissociation (HCD) at a collision energy of 27%. The data were processed using PEAKS 8.5 (Bioinformatics Solutions, Waterloo, ON) and a Pooideae database. Mass tolerances on precursor and fragment ions were 10 ppm and 0.01 Da, respectively. Variable selected posttranslational modifications were carbamidomethyl, oxidation, deamination and phosphorylation.

Scaffold (version Scaffold_4.8.9, Proteome Software, Inc., Portland, OR) was used to validate MS/MS based peptide and protein identifications. Peptide identifications were accepted if they could be established at greater than 97.0% probability to achieve a false discovery rate (FDR) less than 1.0% by the peptide prophet algorithm [36] with Scaffold delta-mass correction. Protein identifications were accepted if they could be established at greater than 99.0% probability and contained at least 2 identified peptides. Protein probabilities were assigned by the Protein Prophet algorithm [37]. Proteins that contained similar peptides and could not be differentiated based on MS/MS analysis alone were grouped to satisfy the principles of parsimony. The peptide sequences identified by mass spectroscopy were further analyzed in silico for their potential bioactivity using the BIOPEP database [38].

### 2.10. Statistical Analysis

Each experiment was conducted in triplicate and data expressed as means ± standard deviation. The data were tested by one-way ANOVA and the significant differences between the means for the values were evaluated by Tukey’s test at 5% level of significance. Pearson correlation coefficients and p-values were used to show correlations and significance. Statistical analyses were performed using XLSTAT software (Addinsoft, New York, NY, USA) in Microsoft Excel (Microsoft, Redmond, WA, USA).

## 3. Results and Discussion

### 3.1. Composition of Dehulled Canary Seed, Oat and Wheat Flours

Dehulled canary seed flour protein content ranged from 21.9–22.5% (*w/w*), with no significant difference between the two yellow and brown cultivars. No significant differences were also observed in total carbohydrate and total polyphenol contents (TPC) among the four canary seed cultivars (Table 1). However, the protein content of the canary seed flours was significantly higher (*p* < 0.05) than those of oat (14.3%, *w/w*) and wheat (16.35%, *w/w*) flours (Table 1), thereby exceeding most commonly consumed cereal grains, whose protein content generally ranges between 10% to 15% of the dry grain [11]. Interestingly, Snowbird wheat flour (0.65 mg FAE/g sample) contained about 2-fold less total polyphenol content (TPC) than canary seeds (1.34–1.47 mg/g sample), while Turcotte oat had the highest content (2.04 mg FAE/g sample) of the cereals. Polyphenols are secondary metabolites produced by plants that play roles in defense mechanisms, primarily for protection against ultraviolet radiation [39]. These plant secondary metabolites are also useful as radical scavengers and possess positive biochemical effects against cardiovascular diseases, cancer growth and age-related diseases [40]. Alfieri and Redaelli [41] recently analyzed twenty oat cultivars and reported their soluble phenol content (SPC) ranging from 0.78 to 1.09 mg GAE/g sample. On the contrary, higher values, up to 1.5 mg GAE/g were found in oat grains by Adom and Liu [42], and a mean of 2.1 mg GAE/g was reported by Menga et al. [43]. Literature information on canary seeds is relatively scarce, despite few studies that have investigated the phenolic profiles of hairy canary seed [44,45], as well as the phenolic profiles and antioxidant activities in germinated canary seed [31]. To the best of our knowledge, no data were previously published on these four hairless canary seeds cultivars to date.

### 3.2. Molecular Weight Distribution of Peptides in the 3K Permeates of Cereal Flours Digestates

**Figure 1:**
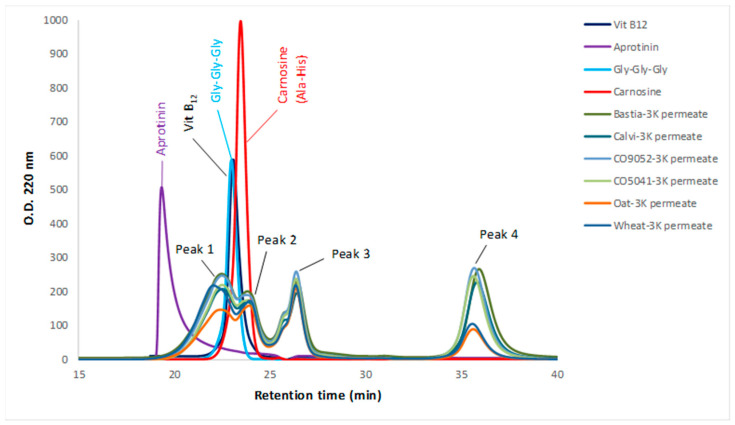
Size-exclusion HPLC profile of canary seed, oat and wheat 3K permeates prepared by subjecting sample flours to in vitro gastrointestinal digestion followed by 3-kDa molecular weight cutoff (MWCO) ultrafiltration. Peaks 1, 2, 3 and 4 represent resolved polypeptides eluted at different retention times than eluted peptides standard named aprotinin, Vit B12, Gly–Gly–Gly and carnosine.

Bioactive peptides are typically characterized by a short chain of 2–20 amino acids [12]; therefore, in vitro digestates were filtered through a 3-kDa MWCO membrane to recover peptides and low molecular weight components with potential higher bioactivity. The soluble protein content in the collected ultrafiltration permeates was significantly higher (*p* < 0.05) in canary seeds (12.9–15.7 mg/mL) than both oat (10.7 ± 1.4 mg/mL) and wheat (10.9 ± 1.0). The SEC-HPLC chromatograms of the 3K permeates are presented in Figure 1. The size-exclusion profile of the four canary seed varieties showed very similar patterns comprising of four well resolved peaks eluted at different retention times of approximately 22.7 min. (peak 1), 23.9 min. (peak 2), 26.4 min. (peak 3) and 35.8 min. (peak 4). As shown in Figure 1, peak 1 had a molecular weight greater than 1.355 kDa corresponding to Vit-B (standard reference). Peak 2 had a molecular weight closer to the dipeptide and tri-peptides corresponding to carnosine (226.23 Da) and Gly–Gly–Gly (189.17 Da), respectively. Peak 3 was of lower molecular weight than the carnosine standard (226.23 Da) since it had a higher retention time (26.4 min.). Peak 4, with the highest retention time, represents a small amount of very low molecular weight peptides that were present in the 3K permeates. In addition, oat and wheat permeates showed four major peaks at similar retention times as canary seed digestates, but with lower peak intensities. No larger aggregates were observed in the filtered digestates, meaning the ultrafiltration step with 3-kDa MWCO membrane was efficient in retaining higher molecular weight components.

### 3.3. Bioactivity Assays

#### 3.3.1. Antioxidant and Chelation Activity

**Figure 2:**
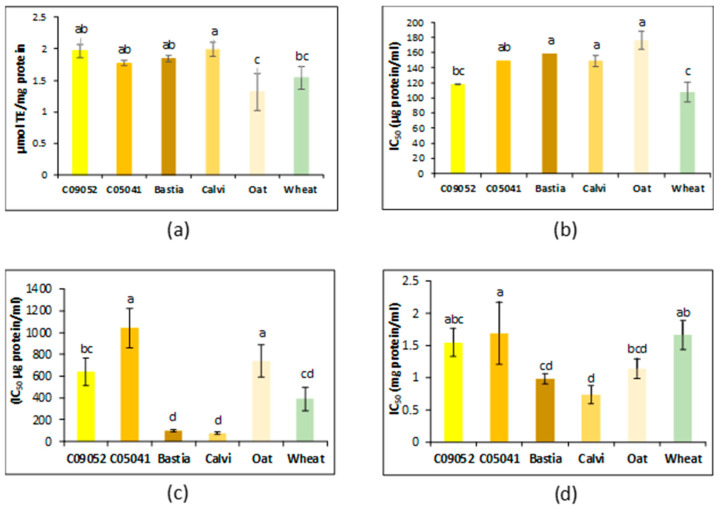
Antioxidant (oxygen radical absorption capacity (ORAC), diammonium salt (ABTS), 2,2-diphenyl-1-picrylhydrazyl (DPPH)) and iron chelation (Fe^2+^) assays of canary seed, oat and wheat 3K permeates prepared following flours in vitro gastrointestinal digestion and 3-kDa MWCO ultrafiltration. (**a**) ORAC, (**b**) ABTS, (**c**) DPPH, (**d**) Fe^2+^ inhibition. IC_50_: inhibitor concentration that inhibits enzyme activity by 50%. TE: Trolox equivalents. Different letters represent statistical difference according to the Tukey test with a confidence level of 95%. In each case the bar is the average of three replicates and standard deviation is indicated.

The potential antioxidant properties of digested cereal 3K permeates was assessed using in vitro studies including a mix of different mechanisms of action: antioxidant in vitro hydrogen atom transfer (HAT) tests which included the ORAC and ABTS assays and in vitro single electron transfer (SET) tests which included DPPH and iron chelating assays [46,47]. Data from the conducted assays (ORAC, DPPH, ABTS) demonstrated different antioxidant profiles for the studied cereal varieties (Figure 2a). For the ORAC assay, the antioxidant activity among canary seed was not significantly different (*p* > 0.05) and ranged from 1.77–1.99-μmol TE/mg protein in the 3K permeate. The Calvi cultivar showed the highest ORAC value overall (1.99-μmol TE/mg) and was significantly (*p* < 0.05) higher than wheat (1.54-μmol TE/mg). Oat had significantly the lowest antioxidant value (1.31-μmol TE/mg). The ORAC values for canaryseeds are higher than what has been reported for the glutelin fraction from cacao seeds (0.28 μmol TE/mg) [48]. However, diversity in methods, sample preparation and composition, reaction conditions and results quantification make extremely difficult the comparison of antioxidant data from different studies. This is particularly true for cereals where research connecting cereal grains with bioactivities after gastrointestinal digestion remains limited. Thus, after an in vitro gastrointestinal digestion, a superior ORAC value has been reported for a < 5 K MWCO digest fraction from quinoa (2.72-μmol TE/mg protein) [49].

For the ABTS assay, lower calculated IC_50_ values indicate higher antioxidant activity. As shown in Figure 2b, by this assay, the yellow C09052 canary seed cultivar and wheat had the highest activity with IC_50_ values of 117.5 μg/mL and 107.8 μg/mL, respectively (*p* > 0.05). While those of Calvi, Bastia, C05041 and oat were not significantly different (*p* > 0.05) from one another. Recently, Valverde et al. [19] reported the antioxidant capacity of soaked (in water for 12 h) hairy canary seed protein fractions (obtained by Osborne fractionation) that were digested in vitro. Their results indicated that digested albumin and prolamin peptides showed the best IC_50_ values, 133.2 and 181.6 μg/mL, respectively (*p* < 0.05); while globulins and glutelins showed high IC_50_ values. The ABTS inhibition activity of canary seeds was also higher than the reported IC_50_ values for chia seed prolamin (161.5 μg/mL) and glutelin (184.7 μg/mL) fractions [29], as well as 0.5–3K MWCO peptides from wheat (174.64 μg/mL) and mung bean (248.97 μg/mL) [50], but significantly lower than the prolamin fraction from red beans (60 μg/mL) [51].

The DPPH assay results (Figure 2c) showed that the brown canary seed cultivars (Calvi and Bastia) have the highest antioxidant activity (77.9–96.4 μg/mL), whereas the yellow C05041 (1043.5 μg/mL) and oat (742.3 μg/mL) had the lowest activity. The IC_50_ values for the DPPH inhibition activity of the brown Bastia (96.4 μg/mL) and Calvi (77.9 μg/mL) 3K permeates were comparable to results reported for chia seed albumin (124.4 μg/mL) and globulin (74.7 μg/mL) protein digestates [29], black bean protein hydrolysate (96.2 μg/mL) purified by ultrafiltration (<4 kDa) [52] and for the prolamin fraction of hairy canary seeds soaked for 12 and 24 h (114.1 and 89.7 μg/mL, respectively) [19]. Both brown canary seed cultivars also had the highest chelation activity (Fe^2+^ inhibition) with IC_50_ values of 0.73 and 0.98-mg/mL for Calvi and Bastia, respectively (Figure 2d). This was also in good correlation with their high DPPH activity. Wheat (1.8 mg/mL) and the yellow C05041 (1.9 mg/mL) 3K permeate had the highest IC_50_ values and therefore the lowest chelating activity. The iron chelating activity in the brown canary seed cultivars was higher than values reported for chia seed flour (1.6 mg/mL) [29]. Interestingly, while ORAC and ABTS assays indicate no differences in antioxidant activity between the yellow and brown canary seed varieties, DPPH, and Fe^2+^ inhibition assays showed higher activities for the brown ones. The different assays showed also different trends in antioxidant activity for oat and wheat. This could be due to a number of reasons, including the different mechanisms (SET and HAT) involved in these assays and by which the antioxidant act in the system, and to their efficacy against different radicals [53]. Another reason for the observed disparity could be due to data quantification. Indeed, in the SET-based assays, the results are quantified as an ‘‘endpoint measurement’’ measuring the change in the oxidant UV-Vis absorbance [47] which possibly may not represent a completed reaction and thereby underestimating radical scavenging activities for slow reacting molecules [53,54,55,56]. HAT-based assays on the other hand are quantified based on the kinetic curve between the probe, antioxidant and peroxyl radicals [47]. Moreover, antioxidant molecules present in the different cereal 3K permeates, including peptides and polyphenols may exhibit different affinity to the different radicals.

**Figure 3:**
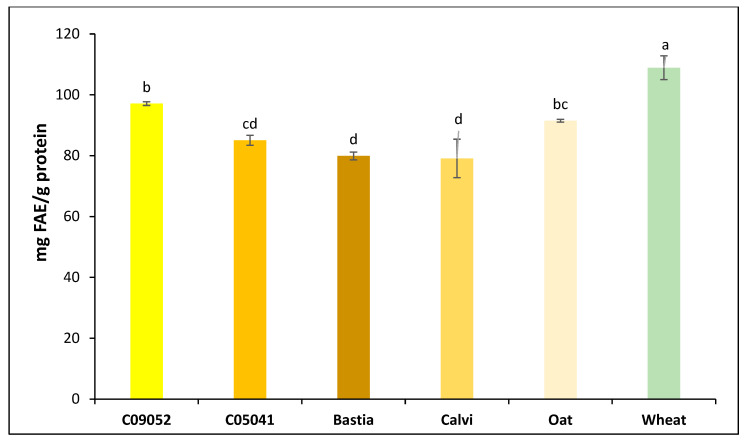
Comparison of total polyphenol content (TPC) of canary seed, oat and wheat 3K permeates prepared following flours in vitro gastrointestinal digestion and 3-kDa MWCO ultrafiltration, Data were expressed as ferulic acid equivalents (FAE). Different letters represent statistical difference according to Tukey test with a confidence level of 95%. Values represent means ± SD (*n* = 3).

According to Kim et al. [57], DPPH activity strongly correlated to the content of phenolic compounds and anthocyanins. However, in our study no correlation was found between DPPH activity and the total polyphenol content (TPC) in the permeates with r2 value of 0.059 (*p* = 0.644). Indeed, the brown canary seed Bastia and Calvi showed the highest DPPH activity with the lowest TPC content (Figure 3), while the wheat permeate exhibited high TPC content (108.9 mg FAE/g protein) and DPPH activity. No significant correlations were also found between the other antioxidant assays and TPC, indicating that phenolics are possibly not the major component responsible for the antioxidant property of canary seed and that bioactive peptides were more accountable for the observed antioxidant activity. This assumption is also supported by SEC-HPLC (Figure 1) results showing higher concentration and resolution of eluted polypeptide fractions from canary seed permeates. Contrarily, wheat permeate had more polyphenols (Figure 3), but less antioxidant activities (ORAC, ABTS and Fe2+ chelating) (Figure 2) due probably to less bioactive peptides present in the permeate as also revealed by SEC-HPLC pattern. As stated in the literature, the antioxidant potency of food components, including polyphenols depends on their bioavailability, which, in turn, depends on their release from the food matrix during digestion [58]. Thus, even though polyphenols content from a specific food source may be high, this may not necessarily be an indication of their contribution to the antioxidant activity, possibly as a result of poor bioavailability. This is further supported by our data and the finding of Zeng et al. [59], who reported a significantly higher bioavailability of the free and bounded phenolic acids of wheat compared to oat, despite a higher content of TPC in oats.

Overall, hairless canary seeds have demonstrated equivalent or superior antioxidant activity than wheat and oat. Brown canary seed cultivars have shown the highest antioxidant activity for the DPPH and iron chelation assays. At this stage, however, it remains difficult to confirm if the antioxidant activity in the digested flours is primarily from the peptides present or if there was a synergistic effect with the cereal polyphenols or other components. Indeed, other studies have also underlined soluble fiber as an additional bioactive compound in cereals. In the colon, fiber is fermented and some bioactive compounds, which have antioxidant activity, are released [60]. From here the importance of multiple test assays to best represent the overall antioxidant activity of a plant, encompassing different antioxidant mechanisms, compound polarity, rate of reaction, and so forth [61].

#### 3.3.2. Antihypertensive Activity

**Figure 4:**
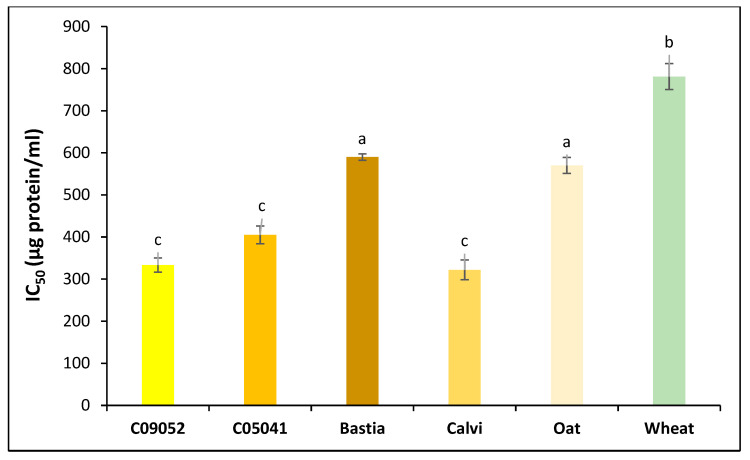
Antihypertensive activity (based on ACE inhibition) of canary seed, oat and wheat 3K permeates prepared following flours in vitro gastrointestinal digestion and 3-kDa MWCO ultrafiltration. IC_50_: inhibitor concentration that inhibits enzyme activity by 50%. Different letters represent statistical difference according to Tukey test with a confidence level of 95%. Values represent mean ± SD (*n* = 3).

The antihypertensive activity of digested cereal 3K permeates was investigated using hippuryl-L-histidyl-L-leucine (HHL) as a substrate. Peptides with high antihypertensive activity can inhibit the ACE enzyme activity and limit the release of hippuric acid (HA). Preliminary tests were first conducted to optimize and determine the appropriate concentration of ACE enzyme required to convert totally the HHL (substrate) into HA, a byproduct of the reaction. The results (Figure 4) show no significant difference (*p* > 0.05) in the antihypertensive activity between the C09052, C05041 and Calvi canary seed cultivars as demonstrated by their respective IC_50_ values of 333.4 μg/mL, 405.0 μg/mL and 321.9 μg/mL. However, the brown Bastia cultivar had a significantly (*p* < 0.05) higher IC_50_ value (589.9 μg/mL) than the other canary seeds cultivars, and hence lower ACE inhibition activity. Similarly, Estrada-Salas et al. [18] reported an ACE inhibition IC_50_ of 332-μg/mL for hairy canary seed peptides, whereas Valverde et al. [19] reported a higher IC_50_ of 217-μg/mL for the prolamin fraction of canary seed flour peptides. The ACE inhibitory activity determined in this study for the yellow C09052 and C05041 and brown Calvi cultivars was comparable to IC_50_ values reported for peptides from chia seed albumin (377 μg/mL) and globulin (339 μg/mL) [29], potato tuber (360 μg/mL) [62] and flaxseed (400 μg/mL) [63]. The IC_50_ of Bastia (589.9 μg/mL) and oat (570.1 μg/mL) digestates were not significantly different (*p* > 0.05); however, wheat digestates exhibited the highest IC_50_ (781.2 μg/mL) revealing the lowest antihypertensive activity. In addition to peptides, other plant components have also been reported to possess in vitro ACE inhibition activity including polyphenols, hydrolysable tannins, flavonoids, fatty acids, oligosaccharides and peptides [64], and which could possibly contribute to the observed ACE inhibition activity.

#### 3.3.3. Antidiabetic Activity

**Figure 5:**
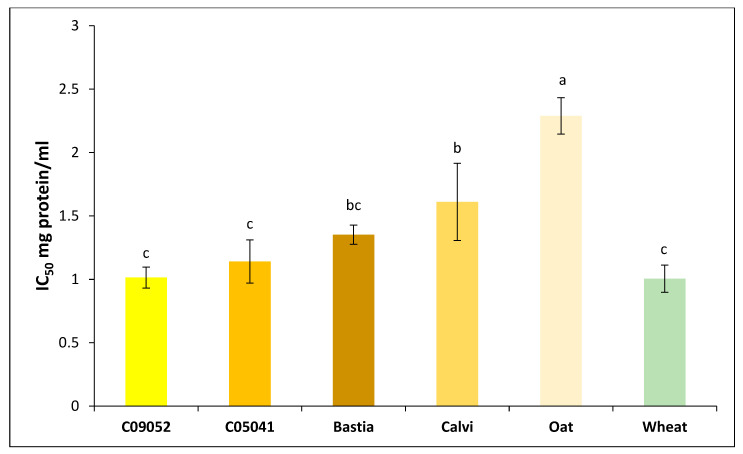
Antidiabetic activity (based on DPP-IV inhibition) of canary seed, oat and wheat 3K permeates prepared following flours in vitro gastrointestinal digestion and 3-kDa MWCO ultrafiltration. IC_50_: inhibitor concentration that inhibits enzyme activity by 50%. Different letters represent statistical difference according to Tukey test with a confidence level of 95%. Values represent mean ± SD (*n* = 3).

The antidiabetic activity of the different digested cereals 3K permeates was assessed through the inhibition capacity of DPP-IV enzyme and determination of IC_50_ values. The results (Figure 5) showed no significant difference (*p* > 0.05) among canary seed cultivars C09052 (IC_50_ value of 1.01 mg/mL), C05041 (IC_50_ value of 1.14 mg/mL), Bastia (IC_50_ value of 1.35 mg/mL) and wheat (IC_50_ value of 1.01 mg/mL), permeates except for the brown Calvi, which exhibited lower DPP-IV inhibition capacity (IC_50_ value of 1.61 mg/mL). Among all studied cereals, oat permeate exhibited the lowest DPP-IV inhibition capacity with IC_50_ value of 2.29-mg/mL (*p* < 0.05). Estrada-Salas et al. [18] reported a maximum inhibition of 43.4% at a peptide concentration of 1.4-mg/mL for hairy canary seeds. The IC_50_ values determined in this study for yellow canary seeds and the brown Bastia cultivar (1.01–1.35 mg/mL) are similar to those reported for amaranth peptides (1.1 mg/mL) [34], germinated soybean peptides (1.49 mg/mL) and oat flour (0.99 mg/mL) [65], while being superior to those reported for barley (3.91 mg/mL) and buckwheat (1.98 mg/mL) flours [65].

Similar to antioxidant and antihypertensive bioactivities, different biomolecules from plants were reported to possess DPP-IV inhibition capacity, including peptides, phenolics and flavonoids [66], which could possibly account for the observed bioactivity. Overall, this study revealed that among the four studied canary seed cultivars, the yellow C09052 and the brown Calvi demonstrated excellent overall biologic activities. In particular, these two cultivars showed superior antihypertensive activity compared to the studied oat and wheat varieties. Since the yellow C09052 is one of the newly developed and less studied canary seed cultivar, it was selected for further investigation of the peptides responsible for the ACE inhibition activity.

### 3.4. Peptide Fractionation and Measurement of ACE Inhibitor Activity

**Figure 6:**
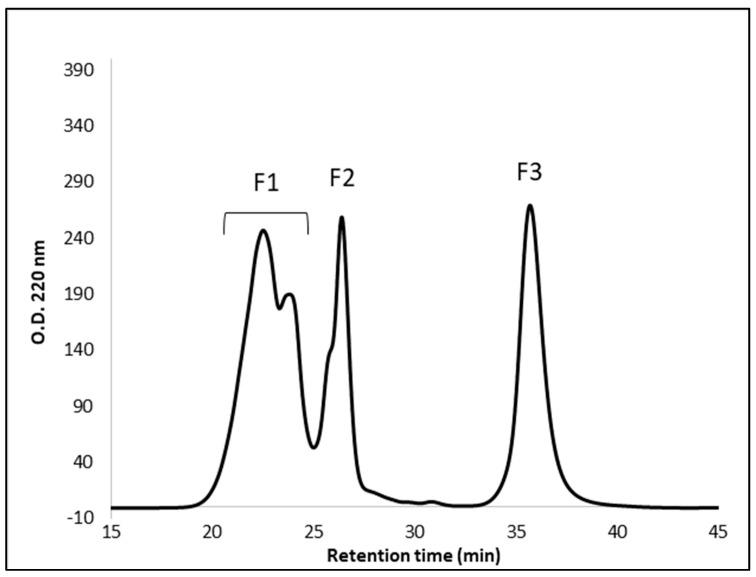
Size-exclusion chromatogram of fractionated canary seed cultivar C09052 3K permeate. F1, fraction 1; F2, fraction 2; F3, fraction 3.

**Figure 7:**
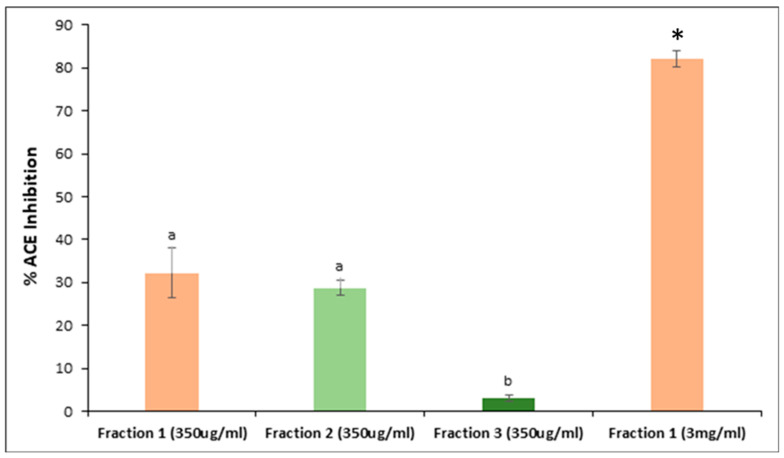
Antihypertensive activity (based on angiotensin converting enzyme (ACE) inhibition) of collected peptide fractions from C09052 canary seed 3K permeate following SE-HPLC separation. Different letters represent statistical difference according to Tukey test with a confidence level of 95%. Values represent mean ± SD (*n* = 3). * Increasing concentration to 3 mg/mL increased %ACE inhibition of fraction 1.

The peptides in the 3K permeate of the yellow C09052 canary seed flour digestate were further fractionated by SEC-HPLC, and the ACE inhibitory activity of each fraction was investigated. The size-exclusion chromatogram of the C09052 permeate is presented in Figure 6, showing the presence of 4 peaks with retention times of approximately 22.7, 26.4 and 35.8 min, respectively. Due to their poor separation, peak 1 and peak 2 were combined as one fraction and the four peaks were designated as F1 (peak 1 and 2), F2 (peak 3) and F3 (peak 4) fractions. F1 and F2 fractions had the highest ACE inhibition activity of 32.2% and 28.8%, respectively (*p* > 0.05). F3, with the highest retention time, had very low activity (3.1%) and protein content, corresponding to smaller molecular weight components that could possibly be assigned to single amino acids or small molecules (solvent, salts) with no ACE inhibition activity. Because the protein content of F3 was relatively small, the ACE inhibition assay was first performed at a peptide concentration of 350 μg/mL for each fraction. At lower protein concentration (350 μg/mL), only 30% of the ACE enzyme was inhibited by F1 and F2. However, when a higher concentration of peptides from F1 was used for the assay (3.0 mg/mL), the ACE inhibitory activity increased to 82.1%, as shown in Figure 7. Interestingly, before fractionation of the C09052 canary seed hydrolysate, the whole hydrolysate showed 50% inhibition at a concentration of 333.4 μg/mL (Figure 4). However, after fractionation, when individual fractions were tested at similar protein concentration of 350 μg/mL, lower ACE inhibitory activity was obtained for all fractions (3–32%) (Figure 7). This loss of activity after fractionation could be due to reduced synergistic interactions between peptides from different fractions, and/or the removal of other constituents in the hydrolysate which could also contribute to ACE inhibitor activity.

### 3.5. Peptide Identification and Potential Bioactivity

#### 3.5.1. Peptide Identification

Tandem MS analysis lead to the identification of 46 peptides in the F1 size-exclusion fraction. The parent protein of each identified peptide is represented in Table 2. Currently, no proteomic database exists for canary seeds and so the identified peptides were analyzed using a Pooideae database, which includes proteins from cereals such as oat, wheat, barley and rye. The peptides were attributed to 18 different parent proteins in total. Canary seeds have been extensively compared to wheat, however, 14 of the 18 proteins identified were of barley origin, indicating canary seeds may contain several proteins similar or identical to those of barley. The shortest and longest peptides were comprised of 8 and 30 amino acids, respectively, with molecular weights ranging from 913.57 Da to 3185.61 Da.

Some peptides (such as AVFPSIVGRPR, FPSIVGRPR, VFPSIVGRPR and GYSFTTTAER) are redundant and present in more than one protein. The redundant peptides are found in proteins with related functions, such as both predicted proteins and uncharacterized proteins from barley. All three proteins are part of the actin cytoskeleton, filamentous proteins involved in cellular organelle and cytoplasmic transportation, which explains why they consist of several identical peptides [67]. Other proteins identified contain unique canary seed peptides but have related functions in the plant. The ATP synthase subunit alpha protein from barley and the ATP synthase subunit beta protein from goat grass play crucial roles in photosynthesis by producing ATP (adenosine triphosphate) and contain unique peptides GIRPAINVGLSVSR and IGLFGGAGVGK, respectively, from canary seeds [68]. As core components of the nucleosome, Histone H4 from einkorn and Histone H2A from wheat fold DNA to form and shape the chromatin [69].

#### 3.5.2. In Silico Prediction of Bioactive Peptide Activity

Table 3 shows the potential bioactivity of the peptides identified by tandem MS. All 46 unique peptides that were identified had potential ACE inhibitor and DPP-IV inhibitor activity and 20 among them had potential antioxidant activity, as confirmed by our in vitro bioactivity testing. Interestingly, 22 peptides had potential hypotensive activity as renin inhibitors. Renin and ACE, two key enzymes in the renin-angiotensin aldosterone system, regulate mammalian blood pressure; renin first converts angiotensinogen to angiotensin 1 which is in turn catalyzed by ACE to angiotensin II, a very powerful vasoconstrictor that simultaneously induces aldosterone secretion, causing increased sodium retention [70]. Although most studies report antihypertensive activity in terms of ACE inhibition, several studies show plant proteins have demonstrated renin inhibitor activity, including canola [71], hempseed [72], lima bean [73], flaxseed [74] and rapeseed [75].

In addition, many of the identified peptides had predicted bioactivities that have not yet been demonstrated in canary seeds, including antiamnestic, antithrombotic, opioid, neuroprotective and immunostimulating activities (Table 3). Of particular interest is the identification of prolyl endopeptidases (PEP’s) inhibition activity. PEP are a group of serine proteases that cleave internal proline residues of peptides, thereby degrading active peptides, hormones and neuropeptides that aid in preventing neurological diseases including Alzheimer’s disease, amnesia, depression and schizophrenia [76,77]. PEP inhibitors are therefore of interest as potential treatments for neurodegenerative disorders. Most biologically active hormone peptides and neuroprotective peptides contain at least one internal proline residue [78]. Five of the identified peptides in canary seed possessed potential antiamnestic activity as PEP inhibitors and each peptide contained at least one proline residue. Most notably is the peptide VGINYQPPTVVPGGDLAK, which contains 3 internal proline residues. The antiamnestic activity of this peptide is most likely higher than in the other 4 peptides which only contain 1 internal proline residue. All five peptides with antiamnestic activity consisted of amino acid sequences PG and/or GP, corresponding to amino acids Pro–Gly and Gly–Pro, respectively. Other identified peptides contain one or more internal proline residues, but did not possess any potential antiamnestic activity because they did not have the PG and/or GP amino acid sequence, however, several of these peptides contain more than one proline residues and could still demonstrate antiamnestic activity.

Furthermore, 5 peptides that demonstrated potential antiamnestic activity also had antithrombotic activity. Antithrombotic peptides reduce venous platelet aggregation and coagulation, thereby helping to control and prevent cardiovascular disease incidence [79]. Of these 5 peptides, FPGQLNADLR, VGINYQPPTVVPGGDLAK and QEYDESGPSIVHR were isolated and identified from enzymatic blue mussel digestates and have demonstrated anticoagulant activity in silico. Furthermore, the peptide QEYDESGPSIVHR was also isolated and identified in olive oil and exhibited anticoagulant activity in vitro [80,81]. Other cereals demonstrating antithrombotic activity include amaranth [82,83], oat, barley and buckwheat [84].

Two canary seed peptides with similar sequences (DLYANTVLSGGTTMYPGIADR and KDLYANTVLSGGTTMYPGIADR) portrayed excellent overall activity including antiamnestic, antithrombotic, opioid, antioxidant and hypotensive activity. Only these 2 peptides had potential opioid activity due to the peptide sequence YPG (Tyr–Pro–Gly). Wheat and soybeans have been studied extensively since they contain many peptides with opioid activity. These exogenous opioid peptides structurally resemble endogenous opioid peptides and interact with opioid like receptors, positively effecting regulatory functions in the central nervous system and gastrointestinal digestion [85,86]. Bioactive peptides with opioid activity usually contain a Tyr–Pro sequence and many opioid peptides from wheat, called exorphins, also contain Gly residues, which coincides with the bioactive sequence Tyr–Pro–Gly that was found in 2 canary seed peptides [87]. Using an in silico approach, Garg, Apostolopoulos, Nurgali and Mishra [88] found the same Tyr–Pro–Gly peptide present in wheat gluten and determined it had opioid activity in vitro. Three peptides from canary seed contained the amino acid sequence GQ (Gly–Gln), giving them potential neuro activity. The endogenously produced dipeptide Gly–Gln has several biological functions in the body, including inducing lymphocytosis, enhancing the activity of natural killer cells, and helping regulate cardiovascular and hypotension functions, among others [89]. However, the neuropeptide activity of Gly–Gln has not been reported in cereals or other food sources to date.

Moreover, three of the identified canary seed peptides had immunostimulating activity from the peptide sequences Gly–Val–Met, Gly–Phe–Leu, and Gly–Leu–Phe (Table 3). Immunostimulating peptides aid the host defense system in several ways, including generating immune cells, support macrophage phagocytosis, increase antibody synthesis and inactivate inflammatory compounds, among others [90]. Among plant proteins, soybeans are known to possess a strong immunostimulating peptide termed ‘soymetide’; a peptide consisting of 13 amino acids (MITLAIPVNKPGR) from its 7S globulin protein [91]. The soymetide peptide does contain amino acids glycine, valine and methionine, but not in the same sequence as canary seed. Silva-Sánchez et al. [92] identified a biopeptide from amaranth protein with the sequence Gly–Phe–Leu that had immunomodulating activity. Although Gly–Leu–Phe and Gly–Val–Met peptides were not found in cereals, they have been identified as immunostimulating peptides from milk sources [93,94,95].

The peptide bioactivity and potential bioactivity profiles were determined in vitro for canary seeds. The biologic potential of a specific peptide in vivo depends primarily on its ability to remain intact until its arrival at the objective organ [96]. Bioactive peptides must first be released from their parent protein during gastrointestinal digestion and remain intact or even hydrolyzed further to retain their bioactivity. Depending on their amino acid composition, some peptides are less digestible than others. Because proline is a secondary amino acid, it requires selective digestive proteases and enzymes to hydrolyze peptide bonds at proline residues, therefore, bioactive peptides containing proline residues are resistant to digestion and protect the active peptide from enzymatic degradation [78]. Nonetheless, some in vitro studies show that resistance to enzymatic hydrolysis during digestion can either increase or decrease a peptide’s bioactivity [96]. Twenty four of the identified peptides in canary seeds possess internal proline residues, potentially making them more resistant to digestion by proteolytic enzymes, which in turn, could enhance or reduce the bioactive effects of the seeds. However, the positive health effects of canary seed proteins have not yet been determined in vivo.

## 4. Conclusions

The in vitro bioactive properties of the low molecular weight (<3 kDa) components of novel yellow and brown hairless canary seed flour in vitro gastrointestinal digestates were evaluated and compared to those of oat and wheat. This approach, although making data interpretation more complex, allowed for the bioactivity assessment of the bioavailable fraction of the cereal flour digestates, while accounting for matrix effect as well as interactions (synergistic or antagonistic) between the various compounds present in the permeates. This being generally disregarded when isolated individual components are assessed [97]. Nonetheless, further research is necessary to investigate the role and identity of each component in the expressed canary seed biologic activities.

Overall, this study indicates that hairless canary seed have antioxidant, chelating, antihypertensive and antidiabetic activity equivalent or superior to the common cereals—oats and wheat. The antihypertensive activity of hairless canary seed was especially high, particularly for the yellow C09052 and the brown Calvi cultivars. Further fractionation and investigation of the yellow canary seed C09052 cultivar allowed the identification of forty six peptides responsible for the ACE inhibitor activity. These peptides were identified belonging to 18 different proteins in the Pooideae subfamily, the majority homologous to proteins from barley origin. In silico analysis of the potential bioactivity shows that all 46 identified peptides had ACE inhibitor and DPP-IV inhibitor activity and 20 had antioxidant activity, which have been validated by in vitro studies. In addition, other peptides had potential antiamnestic, antithrombotic, hypotensive and opioid/neuro activity, which deserve further investigation. Chronic disease, such as heart disease, cancer and diabetes, is a major health concern in society today. This study has demonstrated the potential positive health promoting effects of hairless canary seeds which can be used as novel functional food or ingredient to help in the management of chronic disease, particularly cardiovascular disease.

## Figures and Tables

**Table 1:** Proximate composition of different dehulled hairless canary seeds, oat and wheat flours.

Cereal Cultivars	Proximate Analysis of Flours					
	Ash (%) d.b. ^1^	Moisture (%)	Oil (%) d.b.	Protein (%) d.b.	Total Carbohydrate (%) d.b.	Total Polyphenol (TPC) mg FAE/g
Bastia Brown	2.46 ± 0.03 ^a^	8.94 ± 0.62 ^b^	6.39 ± 0.16 ^b^	22.33 ± 0.26 ^a^	59.88 ± 0.90 ^c^	1.44 ± 0.03 ^b^
Calvi Brown	2.44 ± 0.06 ^a^	8.29 ± 0.16 ^b^	6.29 ± 0.14 ^b^	21.55 ± 0.10 ^a^	61.44 ± 0.38 ^c^	1.42 ± 0.01 ^b^
C05041 Yellow	2.43 ± 0.02 ^a^	8.90 ± 0.26 ^b^	6.03 ± 0.35 ^bc^	21.53 ± 0.25 ^a^	61.11 ± 0.65 ^c^	1.34 ± 0.06 ^b^
C09052 Yellow	2.44 ± 0.08 ^a^	8.58 ± 0.11 ^b^	5.61 ± 0.04 ^c^	21.97 ± 0.56 ^a^	61.41 ± 0.38 ^c^	1.47 ± 0.01 ^b^
Oat (Turcotte)	2.14 ± 0.01 ^b^	7.08 ± 0.10 ^c^	8.44 ± 0.06 ^a^	14.30 ± 0.12 ^c^	65.19 ± 0.59 ^b^	2.04 ± 0.15 ^a^
Wheat (Snowbird)	1.97 ± 0.02 ^c^	10.70 ± 0.47 ^a^	1.26 ± 0.01 ^d^	16.35 ± 0.07 ^b^	70.60 ± 0.57 ^a^	0.65 ± 0.05 ^c^

Means ± standard deviation in a column with different uppercase letters are significantly different at *p* < 0.05 (*n* = 3); ^1^ d.b.: calculated on a dry base. TPC: mg of ferulic acid equivalents per g of sample.

**Table 2:** Identified peptides and parent proteins in F1 from in vitro digestate of yellow C09052 hairless canary seed proteins.

Protein Peptide Sequence	MW (Da)	Protein Accession Number	Organism
Predicted protein	41,787.00	F2D4P0_HORVV	*Hordeum vulgare* subsp. *vulgare* (barley)
AVFPSIVGRPR	1197.71		
DLYANTVLSGGTTMYPGIADR	2214.07		
FPSIVGRPR	1027.60		
GYSFTTTAER	1131.53		
HQGVMVGMGQK	1170.57		
IWHHTFYNELR	1514.75		
KDLYANTVLSGGTTMYPGIADR	2342.17		
SYELPDGQVITIGNER	1789.89		
VFPSIVGRPR	1126.67		
WHHTFYNELR	1401.67		
Predicted protein	42,047.70	F2DZG9_HORVV	*Hordeum vulgare* subsp. *vulgare* (barley)
AVFPSIVGRPR	1197.71		
FPSIVGRPR	1027.60		
GYSFTTTAER	1131.53		
HQGVMVGMGQK	1170.57		
IWHHTFYNELR	1514.75		
QEYDESGPSIVHR	1515.70		
VAPEEHPVLLTEAPLNPK	1953.06		
VFPSIVGRPR	1126.67		
WHHTFYNELR	1401.67		
Uncharacterized protein	42,333.10	A0A287LZV9_HORVV	*Hordeum vulgare* subsp. *vulgare* (barley)
AVFPSIVGRPR	1197.71		
FPSIVGRPR	1027.60		
GYSFTTTAER	1131.53		
IWHHTFYNELR	1514.75		
TTGIVMDSGDGVSHTVPIYEGFTLPHAIIR	3182.61		
VFPSIVGRPR	1126.67		
WHHTFYNELR	1401.67		
Tubulin alpha chain	50,071.70	F2E847_HORVV	*Hordeum vulgare* subsp. *vulgare* (barley)
AFVHWYVGEGMEEGEFSEAR	2345.01		
AVFVDLEPTVIDEVR	1700.91		
DVNAAIATIK	1014.58		
QLFHPEQLITGK	1409.77		
TIGGGDDSFNTFFSETGAGK	2006.89		
VGINYQPPTVVPGGDLAK	1823.99		
Elongation factor 1-alpha	50,936.00	A0A287P673_HORVV	*Hordeum vulgare* subsp. *Vulgare* (barley)
IGGIGTVPVGR	1024.61		
LPLQDVYK	974.55		
QTVAVGVIK	913.57		
STTTGHLIYK	1119.60		
THINIVVIGHVDSGK	1587.88		
Tubulin beta chain	50,691.10	F2D8W7_HORVV	*Hordeum vulgare* subsp. *vulgare* (barley)
FPGQLNADLR	1129.60		
IMNTFSVVPSPK	1334.70		
ISEQFTAMFR	1228.60		
KLAVNMVPFPR	1270.73		
LAVNMVPFPR	1142.63		
Uncharacterized protein	71,301.20	A0A287NGJ6_HORVV	*Hordeum vulgare* subsp. *vulgare* (barley)
HGSLGFLPR	982.54		
Tubulin alpha chain	40,676.70	F2DQT3_HORVV	*Hordeum vulgare* subsp. *vulgare* (barley)
DVNAAIATIK	1014.58		
LISQVISSLTASLR	1486.88		
Predicted protein (Fragment)	22,495.80	F2EJ22_HORVV	*Hordeum vulgare* subsp. *vulgare* (barley)
LKFPLPHR	1006.62		
Predicted protein (Fragment)	40,285.70	F2DYG9_HORVV	*Hordeum vulgare* subsp. *vulgare* (barley)
FATEAAITILR	1204.69		
Predicted protein	71,916.00	F2E5M4_HORVV	*Hordeum vulgare* subsp. *vulgare* (barley)
GVPQIEVTFDLDANGILNVSAVDK	2514.29		
T-complex protein 1 subunit eta	27,260.50	A0A287TMA9_HORVV	*Hordeum vulgare* subsp. *vulgare* (barley)
SLHDAIMIVR	1153.64		
Uncharacterized protein	69,103.00	A0A287QLL8_HORVV	*Hordeum vulgare* subsp. *vulgare* (barley)
DAGVIAGINVLR	1196.70		
ATP synthase subunit alpha	55,307.40	A0A1C9ZNX9_HORVS	*Hordeum vulgare* subsp. *Spontaneum* (barley)
GIRPAINVGLSVSR	1437.85		
Histone H4	11,367.70	M7ZMQ6_TRIUA	*Triticum urartu* (Red wild einkorn)
DNIQGITKPAIR	1324.75		
ISGLIYEETR	1179.62		
KTVTAMDVVYALK	1437.80		
MDVVYALK	937.50		
TVTAMDVVYALK	1325.70		
VFLENVIR	988.58		
Uncharacterized protein	59,868.80	A0A1D5UUD3_WHEAT	*Triticum estivum* (Wheat)
ESTLHLVLR	1066.62		
Histone H2A	17,470.80	A0A0C4BKM5_WHEAT	*Triticum estivum* (Wheat)
AGLQFPVGR	943.53		
ATP synthase subunit beta, chloroplastic	53,842.70	A0A075W706_AEGBI	*Aegilops bicornis* (Spach goat grass)
IGLFGGAGVGK	974.56		

**Table 3:** Potential peptide bioactivity of hairless canary seed in vitro digestates using BIOPEP.

Peptide Sequence	Potential Bioactivity								
AVFPSIVGRPR	ACE Inhibitor	DPP-IV Inhibitor							
DLYANTVLSGGTTMYPGIADR	ACE inhibitor	DPP-IV Inhibitor	Antiamnestic	Antithrombotic	Opioid	Antioxidant	Hypotensive		
FPSIVGRPR	ACE inhibitor	DPP-IV Inhibitor							
GYSFTTTAER	ACE inhibitor	DPP-IV Inhibitor					Hypotensive		
HQGVMVGMGQK	ACE inhibitor	DPP-IV Inhibitor						Immunostimulating	Neuropeptide
IWHHTFYNELR	ACE inhibitor	DPP-IV Inhibitor				Antioxidant	Hypotensive		
KDLYANTVLSGGTTMYPGIADR	ACE inhibitor	DPP-IV Inhibitor	Antiamnestic	Antithrombotic	Opioid	Antioxidant	Hypotensive		
SYELPDGQVITIGNER	ACE inhibitor	DPP-IV Inhibitor				Antioxidant			Neuropeptide
VFPSIVGRPR	ACE inhibitor	DPP-IV Inhibitor							
WHHTFYNELR	ACE inhibitor	DPP-IV Inhibitor				Antioxidant	Hypotensive		
QEYDESGPSIVHR	ACE inhibitor	DPP-IV Inhibitor	Antiamnestic	Antithrombotic					
VAPEEHPVLLTEAPLNPK	ACE inhibitor	DPP-IV Inhibitor				Antioxidant			
TTGIVMDSGDGVSHTVPIYEGFTLPHAIIR	ACE inhibitor	DPP-IV Inhibitor				Antioxidant	Hypotensive		
AFVHWYVGEGMEEGEFSEAR	ACE inhibitor	DPP-IV Inhibitor				Antioxidant	Hypotensive		
AVFVDLEPTVIDEVR	ACE inhibitor	DPP-IV Inhibitor							
DVNAAIATIK	ACE inhibitor	DPP-IV Inhibitor							
QLFHPEQLITGK	ACE inhibitor	DPP-IV Inhibitor							
TIGGGDDSFNTFFSETGAGK	ACE inhibitor	DPP-IV Inhibitor					Hypotensive		
VGINYQPPTVVPGGDLAK	ACE inhibitor	DPP-IV Inhibitor	Antiamnestic	Antithrombotic					
IGGIGTVPVGR	ACE inhibitor	DPP-IV Inhibitor							
LPLQDVYK	ACE inhibitor	DPP-IV Inhibitor				Antioxidant			
QTVAVGVIK	ACE inhibitor	DPP-IV Inhibitor							
STTTGHLIYK	ACE inhibitor	DPP-IV Inhibitor				Antioxidant			
THINIVVIGHVDSGK	ACE inhibitor	DPP-IV Inhibitor							
FPGQLNADLR	ACE inhibitor	DPP-IV Inhibitor	Antiamnestic	Antithrombotic			Hypotensive		Neuropeptide
IMNTFSVVPSPK	ACE inhibitor	DPP-IV Inhibitor							
ISEQFTAMFR	ACE inhibitor	DPP-IV Inhibitor					Hypotensive		
KLAVNMVPFPR	ACE inhibitor	DPP-IV Inhibitor							
LAVNMVPFPR	ACE inhibitor	DPP-IV Inhibitor							
HGSLGFLPR	ACE inhibitor	DPP-IV Inhibitor						Immunostimulating	
LISQVISSLTASLR	ACE inhibitor	DPP-IV Inhibitor					Hypotensive		
LKFPLPHR	ACE inhibitor	DPP-IV Inhibitor				Antioxidant	Hypotensive		
FATEAAITILR	ACE inhibitor	DPP-IV Inhibitor					Hypotensive		
GVPQIEVTFDLDANGILNVSAVDK	ACE inhibitor	DPP-IV Inhibitor							
SLHDAIMIVR	ACE inhibitor	DPP-IV Inhibitor				Antioxidant			
DAGVIAGINVLR	ACE inhibitor	DPP-IV Inhibitor					Hypotensive		
GIRPAINVGLSVSR	ACE inhibitor	DPP-IV Inhibitor				Antioxidant	Hypotensive		
DNIQGITKPAIR	ACE inhibitor	DPP-IV Inhibitor				Antioxidant	Hypotensive		
ISGLIYEETR	ACE inhibitor	DPP-IV Inhibitor				Antioxidant			
KTVTAMDVVYALK	ACE inhibitor	DPP-IV Inhibitor				Antioxidant	Hypotensive		
MDVVYALK	ACE inhibitor	DPP-IV Inhibitor				Antioxidant	Hypotensive		
TVTAMDVVYALK	ACE inhibitor	DPP-IV Inhibitor				Antioxidant	Hypotensive		
VFLENVIR	ACE inhibitor	DPP-IV Inhibitor				Antioxidant	Hypotensive		
ESTLHLVLR	ACE inhibitor	DPP-IV Inhibitor				Antioxidant	Hypotensive		
AGLQFPVGR	ACE inhibitor	DPP-IV Inhibitor					Hypotensive		
IGLFGGAGVGK	ACE inhibitor	DPP-IV Inhibitor						Immunostimulating	
